# Complete radiological clearance in bilateral loculated pyopneumothorax managed conservatively

**DOI:** 10.4103/0970-2113.48901

**Published:** 2009

**Authors:** S. K. Verma, Vineet Mahajan

**Affiliations:** *Department of Pulmonary Medicine, Chhatrapati Shahuji Maharaj Medical University, UP, Lucknow, India*

**Keywords:** Pyopneumothorax, loculated, antibiotics

## Abstract

We are reporting a case of bilateral loculated pyopneumothorax in a 30-year-old male patient, who was treated conservatively with antibiotics alone, and had complete radiological clearance.

## INTRODUCTION

Loculated pyopneumothorax can occur as a complication of staphylococcal bacteremia, but even after successful treatment, some residual pulmonary or pleural shadowing may persist.^1^ Complete radiological clearing by conservative management with antibiotics alone is being reported here for the purpose of clinical interest owing to reports on this condition being scanty in the literature.

## CASE HISTORY

A 30-year-old man was admitted to our department with complaints of cough, mucopurulent expectoration, high grade fever, dyspnea, and bilateral chest pain for last 10 days. He was a nonsmoker with no past or family history of tuberculosis. Physical examination revealed a moderately built individual. His pulse was 120/minute, blood pressure 100/70 mm Hg, and respiratory rate 32/minute. There was a swelling on left forearm which was fluctuant and tender to touch. Respiratory system examination revealed decreased movements in bilateral infra-axillary regions. Percussion note was hyper-resonant in axillary and stony dull in bilateral infra-axillary regions. On auscultation, there were decreased breath sounds and amphoric breath sounds in both infra-axillary regions.

**Figure 1:**
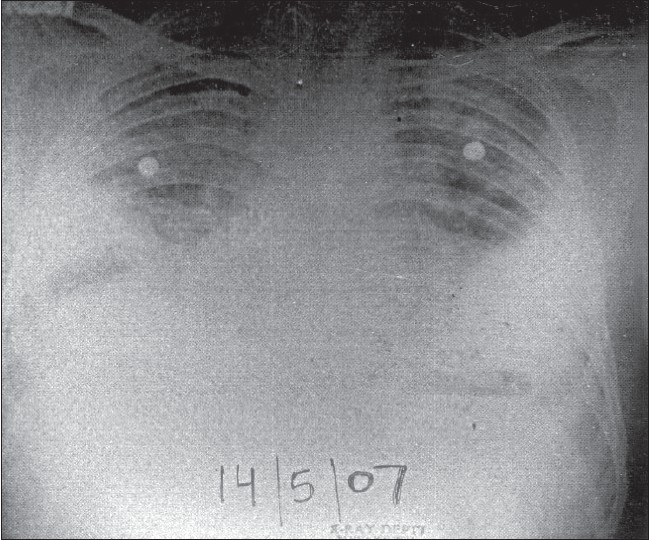
Chest radiograph done three days prior to hospitalization showing nonhomogeneous opacity in right lower zone with ill-defined margins, suggestive of a parenchymal consolidation process, and left-sided pleural effusion

**Figure 2:**
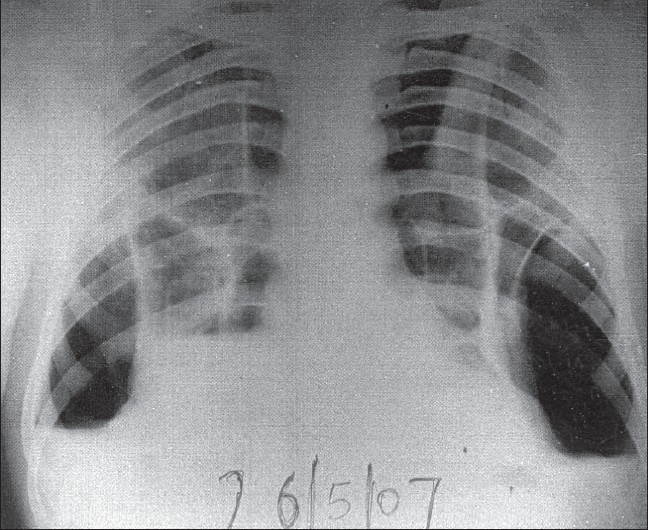
Repeat chest radiograph revealing bilateral air-fluid levels, suggestive of bilateral loculated pyopneumothorax

Laboratory investigations revealed total leukocyte count (TLC) of 27000/mm^3^ with 90% polymorphs. Sputum Gram's stain revealed Gram-positive cocci, but culture grew only commensals. Sputum for acid fast bacilli was repeatedly negative. Biochemical parameters (hepatic and renal function tests) were normal. Serological testing for human immunodeficiency virus was negative. Chest radiograph done three days prior to hospitalization had shown nonhomogeneous opacity in right lower zone with ill-defined margins, suggestive of a parenchymal consolidation process and left-sided pleural effusion [Figure 1]. A repeat chest radiograph revealed bilateral air-fluid levels, suggestive of bilateral loculated pyopneumothorax [Figure 2].

Thick greenish-yellow pus was aspirated from the subcutaneous forearm swelling. Pus smear showed necrotic material with degenerated neutrophils and Gram-positive cocci. *Staphylococcus aureus* grew on culture that was sensitive to piperacillin-tazobactam, amikacin, gentamicin, ciprofloxacin, and cefotaxime.

**Figure 3:**
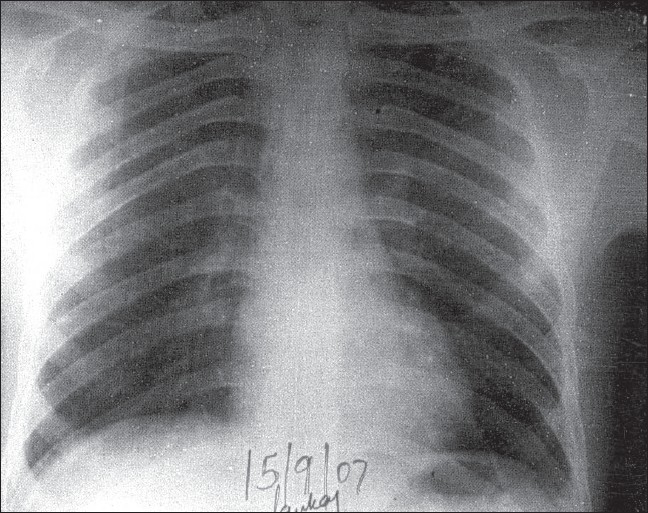
Chest radiograph done after four months is normal

Hence, a diagnosis of bilateral pyopneumothorax complicating staphylococcal bacteremia was made. The patient was managed with parenteral antibiotics (piperacillin-tazobactam 4.5 g, 8 hourly; amikacin 500 mg twice daily; and metronidazole 500 mg, 8 hourly) for two weeks along with symptomatic treatment. The patient became afebrile, TLC became normal, and the forearm abscess resolved. A repeat chest radiograph performed after two weeks showed radiological improvement. The patient was discharged after three weeks. Chest radiograph performed four months later showed complete clearance [Figure 3].

## DISCUSSION

Staphylococcal pneumonia can itself be a complication of a bacteremic illness that may manifest in many ways.^2^ In our case, polymorphonuclear leukocytosis and subcutaneous abscess showing staphylococcus favored the diagnosis of staphylococcal bacteremia. *S. aureus* can gain access to the lung parenchyma by two routes: aspiration of upper respiratory flora and hematogenous spread.^3^ Staphylococcal pneumonia is relatively uncommon, but severe infection is characterized clinically by chest pain, systemic toxicity, and dyspnea. Empyema is a common complication and increases the already considerable morbidity associated with this infection.^3^ Pneumothorax may occur as a result of focal sepsis rupturing through the visceral pleura. This complication is more likely if mechanical ventilation is required or if the patient is taking corticosteroids for some other reason.^4^ When it does occur, it is liable to get complicated by infection in the pleural space, with resultant pyopneumothorax.

There are two basic principles for the successful management of loculated pyopneumothorax complicating pneumonia: (a) control of infection with appropriate antimicrobial therapy, and (b) adequate drainage of pus either by aspiration or tube thoracentesis.^5^ Repeated aspirations as well as tube thoracentesis are painful procedures and increase morbidity. Residual pulmonary or parenchymal shadowing is not uncommon after successful treatment of the staphylococcal pneumonia and its intrathoracic complications.^1^

We conservatively managed the patient with antibiotics and symptomatic treatment, and it lead to complete radiological clearing after four months. This shows that even bilateral loculated pyopneumothorax can be managed conservatively without increasing the morbidity of the patient by repeated aspirations.

## CONCLUSION

The patient can be observed for response of antibiotics, and if there is improvement with antibiotics only, conservative management should be prolonged. Because of the above case study it can be concluded that bilateral loculated pyopneumothorax can be managed conservatively with antibiotics only.
